# Empfehlungen zu Standardkonzentrationen für die kontinuierliche Infusion von Arzneimitteln auf Intensivstationen

**DOI:** 10.1007/s00063-025-01264-x

**Published:** 2025-03-20

**Authors:** L. Kreysing, H. Hilgarth, M. Bodenstein, N. Haake, A. Kaltwasser, J. A. Köck, D. Meyn, R. Riessen, C. Waydhas, I. Krämer

**Affiliations:** 1 Apotheke der Universitätsmedizin Mainz, Johannes Gutenberg-Universität, Langenbeckstraße 1, 55131 Mainz, Deutschland; 2 ADKA Akademie für Krankenhauspharmazie, Bundesverband Deutscher Krankenhausapotheker e. V. (ADKA), Berlin, Deutschland; 3 Klinik für Anästhesiologie Universitätsmedizin Mainz, Johannes Gutenberg-Universität Mainz, Mainz, Deutschland; 4 Praxis für Herz-Kreislauf-Sportmedizin und Perioperative Medizin, Kiel, Deutschland; 5 Akademie der Kreiskliniken Reutlingen gGmbH, Reutlingen, Deutschland; 6 Apotheke des Universitätsklinikums Erlangen, Erlangen, Deutschland; 7 Gesundheit Nordhessen Holding AG, Kassel, Deutschland; 8 Department für Innere Medizin, Universitätsklinikum Tübingen, Tübingen, Deutschland; 9 Klinik für Unfall‑, Hand- und Wiederherstellungschirurgie, Universitätsklinikum Essen, Universität Duisburg-Essen, Essen, Deutschland; 10 Bundesverband Deutscher Krankenhausapotheker e. V. (ADKA), Berlin, Deutschland; 11 Deutsche interdisziplinäre Vereinigung für Intensiv- und Notfallmedizin e. V. (DIVI), Berlin, Deutschland

**Keywords:** Kontinuierliche Infusion, Arzneimitteltherapiesicherheit, Intensivpatienten, Standardkonzentrationen, Evidenzbasierter Entscheidungsprozess, Continuous infusion, Drug therapy safety, Intensive care patients, Standard concentrations, Evidence-based decision-making process

## Abstract

**Hintergrund:**

Die Applikation von Arzneimittelinfusionen in Standardkonzentrationen (SK) und die Steuerung der Dosierung über das Applikationsvolumen bzw. die Applikationsgeschwindigkeit sind geeignete Maßnahmen zur Fehlervermeidung und Verbesserung der Arzneimitteltherapiesicherheit bei Intensivpatienten. Derzeit werden in den deutschen Intensivstationen verschiedene Wirkstoffe in verschiedenen Konzentrationen als Dauerinfusion appliziert.

**Ziel der Arbeit (Fragestellung):**

Ziel dieser ADKA/DIVI-Initiative war die Erstellung einer bundeseinheitlichen Standardkonzentrationsliste (SKL) für die kontinuierliche Infusion von Arzneimitteln auf Erwachsenen-Intensivstationen.

**Material und Methoden:**

Die SKL wurde in einem zweistufigen Verfahren durch eine mandatierte Expertengruppe erstellt. In Stufe 1 wurde die Aufnahme von Wirkstoffen, in Stufe 2 die SK auf Basis von jeweils einer evidenzbasierten Vorschlagsliste und vordefinierten Entscheidungskriterien konsentiert.

**Ergebnisse:**

Die SKL enthält 41 Wirkstoffe mit 49 SK (37 Wirkstoffe je 1 SK, Heparin 2 SK, Epinephrin und Sufentanil 3 SK, Norepinephrin 4 SK) unter Angabe der geeigneten Trägerlösung und der physikalisch-chemischen Stabilität über 24 h.

**Schlussfolgerung:**

Die Empfehlungen zu SK für die kontinuierliche Infusion berücksichtigen die in Deutschland angewandten Wirkstoffe/Arzneimittel zur Behandlung von erwachsenen Intensivpatienten. Die klinikweite Implementierung sollte von einem interprofessionellen Team begleitet werden.

**Zusatzmaterial online:**

Die Online-Version dieses Beitrags (10.1007/s00063-025-01264-x) enthält eine weitere Tabelle über aufgenommene und ausgeschlossene Standardkonzentrationen.

## Abstract (en)

**Background:**

Numerous medications are continuously administered to intensive care patients. To minimize medication errors standardized concentrations and dose adjustment by the infusion rate are recommended. This principle is already widely accepted in German intensive care units (ICUs). However, the active ingredients and concentrations used are still heterogeneous throughout the country.

**Aim:**

The aim of the ADKA/DIVI (Bundesverband Deutscher Krankenhausapotheker/Deutsche Interdisziplinäre Vereinigung für Intensiv- und Notfallmedizin) initiative was to create a nationwide standard for adult continuous intravenous infusions in German ICUs by an expert panel.

**Materials and methods:**

A mandated group of experts developed a nationwide standard concentration list for continuous infusion in a two-stage, consensus-based process. In stage 1, the inclusion of active substances was agreed upon, and in stage 2, the standard concentrations to be recommended were decided upon. The decision-making process was based on predefined decision criteria.

**Results:**

The continuous infusion standard contains 41 active substances with 49 standard concentrations (37 substances with 1 concentration [conc.], heparin 2 conc., epinephrine and sufentanil 3 conc., norepinephrine 4 conc.), along with the appropriate vehicle solution, and the physicochemical stability over maximum 24 h.

**Conclusion:**

The resulting continuous infusion standards are practical and evidence-based and represent a central component for improving medication safety in ICUs. Implementation of the standard list in German ICUs should be facilitated by interdisciplinary teams.

Die Dauerinfusion von Arzneimitteln in Standardkonzentrationen und Steuerung der Dosierung über die Applikationsgeschwindigkeit wird bei Intensivpatienten zunehmend praktiziert, aber in den Kliniken unterschiedlich gehandhabt. Hier wird die von einer mandatierten Expertengruppe entwickelte bundeseinheitliche Standardkonzentrationsliste für Dauerinfusionen vorgestellt.

## Einleitung

Die Verordnung, Zubereitung und Applikation von parenteralen Arzneimitteln, die kontinuierlich als so genannte Dauerinfusion appliziert werden, stellt einen besonders komplexen und risikoreichen Medikationsprozess dar [13, 16]. Konventionell werden Arzneimittel zur Dauerinfusion in Form der Gesamtdosis und teilweise ohne spezifizierende Angaben zu Zubereitung, Volumen, Trägerlösung, Infusionszeit oder Infusionsgeschwindigkeit durch den Arzt verordnet und durchlaufen bis zur Applikation viele potenziell fehlerträchtige Teilprozesse [20]. Die Anwendung von Dauerinfusionen in Standardkonzentrationen (SK) und die Steuerung der Dosierung über das Applikationsvolumen bzw. die Applikationsgeschwindigkeit stellen geeignete Maßnahmen zur Fehlervermeidung und Verbesserung der Arzneimitteltherapiesicherheit (AMTS) dar [8, 14].

In den USA und Großbritannien ist die geschwindigkeitsgesteuerte Dauerinfusion mit SK schon länger praxisüblich [5, 25, 27]. Auch eine deutschlandweit durchführte Umfrage auf Intensivstationen zeigte eine hohe Akzeptanz von geschwindigkeitsgesteuerten Dauerinfusionen mit SK, wenngleich die eingesetzten Wirkstoffe und die gewählten SK unterschiedlich waren [19]. Eine interprofessionell erarbeitete bundesweite Standardkonzentrationsliste (SKL) für Dauerinfusionen kann zur Prozessoptimierung und Erhöhung der AMTS auf Intensivstationen beitragen [19]. Standardkonzentrationen unterstützen zudem die Digitalisierung der Arzneimittelanwendung und die Nutzung von Medikamentenbibliotheken in den Infusions- bzw. Spritzenpumpen.

Ziel dieser ADKA/DIVI-Initiative war die Erstellung einer bundeseinheitlichen SKL für die kontinuierliche Infusion von Arzneimitteln auf Erwachsenen-Intensivstationen.

## Methodik

Die **Expertengruppe** wurde von den Fachgesellschaften Bundesverband Deutscher Krankenhausapotheker e. V. (ADKA) und Deutsche Interdisziplinäre Vereinigung für Intensiv- und Notfallmedizin e. V. (DIVI) mandatiert (02/2022) und setzte sich aus drei Intensivmedizinern, vier Krankenhausapothekern, einer Fachpflegekraft und der Vorsitzenden der DIVI-Sektion „Angewandte Pharmakologie in der Intensiv- und Notfallmedizin“ zusammen.

Die bundesweite SKL für Dauerinfusionen wurde in einem zweistufigen, konsensbasierten Verfahren (nominaler Gruppenprozess) entwickelt. Zunächst wurde über den Einschluss der Wirkstoffe in die SKL (Stufe 1) und daran anschließend über die zu empfehlenden Konzentrationen (Stufe 2) abgestimmt. Dazu wurden die Vorschlagslisten, die Entscheidungskriterien und entscheidungsrelevante Informationen von L. Kreysing und H. Hilgarth an die Mitglieder der Expertengruppe versandt. In beiden Stufen wurden zunächst die Rückmeldungen ausgewertet, eingegangene Kommentare zusammengestellt und der Expertengruppe zur Beratung vorgelegt. In Videokonferenzen wurde über jeden aufzunehmenden Wirkstoff und die zu empfehlenden SK beraten, die Empfehlungen mehrheitlich konsentiert, Begründungen und Ablehnungen protokolliert.

Die Vorschlagsliste für **Stufe 1** umfasste die Wirkstoffe der DIVI-Umfrage [19] und entscheidungsrelevante Informationen (u. a. Dosierung und Art der Anwendung gemäß Punkt 4.2 der Fachinformation). Anhand der in Tab. 1 gelisteten Kriterien stimmte jedes Mitglied der Expertengruppe unabhängig über die Aufnahme in die bundesweite SKL elektronisch mit Ja oder Nein ab. Bei Ablehnung war eine Begründung in Form von vorformulierten Begründungen (Dropdownmenü) oder als Freitext einzufügen.

**Tab. 1:** Entscheidungskriterien zur Konsensfindung in Stufe 1

Nr.	Entscheidungskriterium zur Konsensfindung in Stufe 1
S1‑1	Häufiger Einsatz des Wirkstoffs als Dauerinfusion gemäß Kreysing et al. [19]
S1‑2	Plausibilität einer Dauerinfusion des Wirkstoffes
	Fehlende Plausibilität
	*Hohes Risiko für Nebenwirkungen*
	*Hohes Risiko für Inkompatibilitäten bei Parallelinfusion*
	*Fraglicher/mangelnder Nutzen einer Dauerinfusion*
	*Peridurale Dauerinfusion*
S1‑3	Zulassungsstatus des Wirkstoffs als Dauerinfusion
	Fehlende Zulassung
	*Keine Verfügbarkeit in Deutschland*
	*Dauerinfusion als Off-Label-Use*

Für die **Stufe 2** wurden die in Stufe 1 konsentierten Wirkstoffe um Vorschläge für die SK ergänzt, die den in der DIVI-Umfrage favorisierten Konzentrationen entsprachen [19]. Ergänzt wurden als entscheidungsrelevante Informationen: (I) Stabilität der applikationsfertigen Infusionen über 24 h bei Raumtemperatur (RT) gemäß Datenbankrecherchen (*Fachinformationen, Stabilis-Datenbank* [24]*, Google Scholar, Trissel’s 2 Clinical Pharmaceutics Database (über Lexicomp)* [26]*, PubMed)* und Anfragen bei Arzneimittelherstellern; (II) SKL USA*, ASHP* *Standardize 4 Safety Initiative* [5], (III) SKL UK *Standard Medication Concentrations for Continuous Infusions in Adult Critical Care* [27], (IV) *UCL Hospitals Injectable Medicines Administration Guide* [17], (V) *Medikamenten-Pocket Intensivmedizin: Perfusoren und Spritzenpumpen* [6]; (VI) Verfügbarkeit des Arzneimittels in der SK als Fertigarzneimittel oder als Eigenherstellung der Krankenhausapotheke. Anhand der Kriterien S2‑1 bis S2‑8 (Tab. 2) stimmte jedes Mitglied der Expertengruppe schriftlich über die Aufnahme der vorgeschlagenen SK analog zu Stufe 1 ab.

**Tab. 2:** Entscheidungskriterien zur Konsensfindung in Stufe 2. (Nach [5, 6, 17, 19, 27])

Nr.	Entscheidungskriterium zur Konsensfindung in Stufe 2
S2‑1	Bevorzugt Volumen-sparende Konzentrationen (Standardvolumen 50 mL) festlegen [5]
S2‑2	Bevorzugt Konzentrationen festlegen, die als Fertigarzneimittel oder eigenhergestellt durch die Krankenhausapotheke applikationsfertig zur Verfügung stehen [5]
S2‑3	Möglichst nur eine Konzentration pro Wirkstoff festlegen [5]
S2‑4	Konzentrationen festlegen, die sicher, effizient aus den zu Verfügung stehenden Fertigarzneimitteln zubereitet werden können [5]
S2‑5	Akzeptanzrate der Standardkonzentration in der DIVI-Umfrage [19]
S2‑6	Belegte physikalisch-chemische Stabilität des Arzneimittels in der gewählten Konzentration im Primärpackmittel
S2‑7	Abgleich mit Standardkonzentrationen in internationalen Empfehlungen [5, 27]
S2‑8	Abgleich mit Empfehlungen zur Applikation in ausgewählter Literatur [6, 17]

Mit den Ergebnissen der Stufe 1 (Wirkstoffe) und Stufe 2 (Standardkonzentrationen) wurde, ergänzt um Standardmenge/Standardvolumen, Standardträgerlösung, physikalisch-chemische Stabilität, eine nationale SKL für Dauerinfusionen erstellt. Diese wurde gemäß Beschluss des DIVI-Präsidiums allen DIVI-Mitgliedern zur Kommentierung zur Verfügung gestellt (10.07.2023–10.08.2023). Eingehende Anfragen und Anmerkungen wurden von der Expertengruppe geprüft und ggf. Änderungen vorgenommen. Die finale SKL wurde den Gremien der beteiligten Verbände zur Freigabe vorgelegt.

## Ergebnisse

**Abb. 1:**
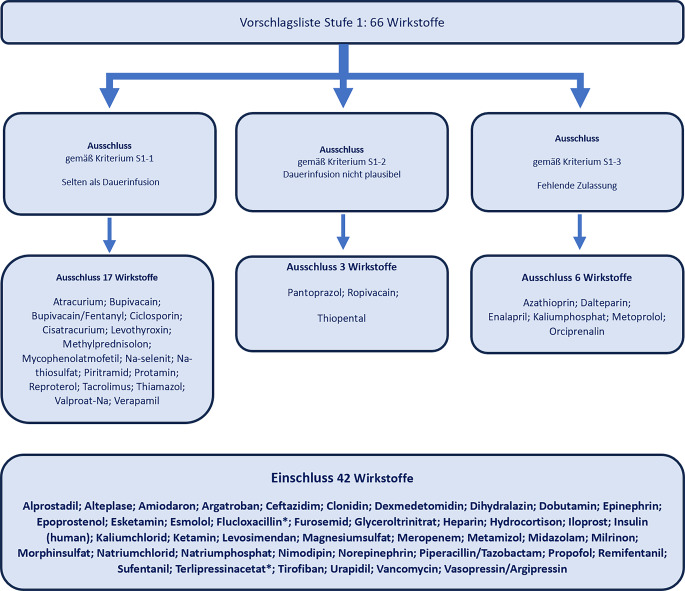
Konsentierte Wirkstoffe gemäß Expertenbeschluss in Stufe 1 (58-mal 7, 5‑mal 6, 2‑mal 5 abstimmende Experten). * zusätzliche Aufnahme gemäß Konsens Expertengruppe

In Stufe 1 enthielt die Vorschlagsliste 66 Wirkstoffe aus der 42 Wirkstoffe und -kombinationen von der Expertengruppe unabhängig ausgewählt wurden. In der anschließenden Expertendiskussion wurden fünf Wirkstoffe (Ciclosporin, Piritramid, Ropivacain, Thiopental, Valproat) ausgeschlossen (Abb. 1). Ausschlussgründe waren zu 65 % (17/26) der seltene Einsatz des Wirkstoffs als Dauerinfusion (Kriterium S1-1), zu 12 % (3/26) fehlende Plausibilität für die Dauerinfusion (S1-2) sowie zu 23 % (6/26) fehlende Zulassung in Deutschland bzw. fehlende Zulassung als Dauerinfusion (S1-3). Anstelle der ursprünglichen Vasopressin-Analoga wurden die Wirkstoffe Vasopressin/Argipressin und Terlipressin getrennt aufgenommen. Terlipressin ist ausdrücklich zur kontinuierlichen Applikation empfohlen, weil dabei weniger schwerwiegende Nebenwirkungen auftreten als bei Bolusapplikation (Rote-Hand-Brief 12/2022) [9]. Die Wirkstoffe Ketamin, Esmolol und Epoprostenol wurden als unverzichtbar eingestuft und beibehalten. Neu aufgenommen wurde Flucloxacillin als häufig bei Intensivpatienten kontinuierlich appliziertes Antibiotikum. Immunsuppressiva wurden nicht aufgenommen, weil diese zumeist auf spezialisierten Stationen angewendet und indikationsspezifisch über unterschiedliche Zeitintervalle appliziert werden.

In Stufe 2 wurden für die 42 Wirkstoffe insgesamt 65 SK vorgeschlagen und der Expertengruppe zur Abstimmung vorgelegt (Tabelle S1 im Online-Zusatzmaterial). Um einer Verwechselung von Ketamin und Esketamin vorzubeugen, wurde Ketamin nachträglich gestrichen. Als ergänzender Hinweis wurde konsentiert, dass z. B. bei Lieferschwierigkeiten von Esketamin (25 mg/mL) mit gleicher Laufrate Ketamin (50 mg/mL) eingesetzt werden kann. Für die nun insgesamt 41 Wirkstoffe wurden 49 SK konsentiert.

Für 37 Wirkstoffe (90 %) konnte jeweils eine (einzige) geeignete SK konsentiert werden. Für vier Wirkstoffe wurde mehr als eine SK konsentiert, um die verschiedenen Indikationen mit unterschiedlichen Dosierungen adäquat abzubilden und zu niedrige Laufraten oder zu häufige Spritzenwechsel zu vermeiden. Für Heparin resultierten zwei, für Epinephrin und Sufentanil jeweils drei sowie für Norepinephrin vier SK (Tab. 3).

**Tab. 3:** Empfehlungen zu Standardkonzentrationen für die kontinuierliche Infusion auf Intensivstationen

Wirkstoff	Standardkonzentration	Standardmenge/Standardvolumen	Standardträgerlösung in bevorzugter Reihenfolge	Physikalisch-chemische Stabilität: 24 h, RT, v. a. PP-Spritze *
Alprostadil	0,8 µg/mL	40 µg/50 mL	0,9 % NaCl	Ja
Alteplase	1 mg/mL	50 mg/50 mL	Entfällt	8 h
Amiodaron^1^	21 mg/mL	1050 mg/50 mL	G5 %	Ja
Argatroban	1 mg/mL	50 mg/50 mL	Entfällt	Ja
Ceftazidim	40 mg/mL	2000 mg/50 mL	0,9 % NaCl	Ja
Clonidin	15 µg/mL	750 µg/50 mL	0,9 % NaCl	Ja
Dexmedetomidin	8 μg/mL	400 µg/50 mL	0,9 % NaCl	Ja
Dihydralazin	1 mg/mL	50 mg/50 mL	0,9 % NaCl	Ja
Dobutamin	5 mg/mL	250 mg/50 mL	Entfällt	Ja
				Bei Rosafärbung: kein wesentlicher Aktivitätsverlust
Epinephrin	0,02 mg/mL	1 mg/50 mL	G5 %, 0,9 % NaCl	Ja
Epinephrin	0,1 mg/mL	5 mg/50 mL	G5 %, 0,9 % NaCl	Ja
Epinephrin	0,2 mg/mL	10 mg/50 mL	G5 %, 0,9 % NaCl	Ja
Epoprostenol	2 µg/mL	100 µg/50 mL	0,9 % NaCl	Ja
Esketamin ^2^	25 mg/mL	1250 mg/50 mL	Entfällt	Ja
Esmolol	10 mg/mL	2500 mg/250 mL Infusionsbeutel	Entfällt	Ja
Flucloxacillin	80 mg/mL	4000 mg/50 mL	0,9 % NaCl	Ja
Furosemid	10 mg/mL	500 mg/50 mL	Entfällt	Ja
Glyceroltrinitrat	1 mg/mL	50 mg/50 mL	Entfällt	Ja
Heparin	200 I.E./mL	10.000 I.E./50 mL	0,9 % NaCl	Ja
Heparin	500 I.E./mL	25.000 I.E./50 mL	0,9 % NaCl	Ja
Hydrocortison	2 mg/mL	100 mg/50 mL	0,9 % NaCl, G5 %	Ja
Iloprost	2 µg/mL	100 µg/50 mL	0,9 % NaCl, G5 %	Ja
Insulin (human)	1 I.E./mL	50 I.E./50 mL	0,9 % NaCl	Ja
Kaliumchlorid	1 mmol/mL K^+^/1 mmol/mL Cl^−^	50 mmol/50 mmol/50 mL	Entfällt	Ja
Levosimendan	0,025 mg/mL	12,5 mg/500 mL Infusionsbeutel	0,9 % NaCl für Pulver G5 % für Konzentrat	Ja
Magnesiumsulfat ^3^	0,4 mmol/mL (Mg^2+^-Ionen)	20 mmol/50 mL (Mg^2+^-Ionen)	Entfällt	Ja
Meropenem	20 mg/mL	1000 mg/50 mL	0,9 % NaCl	Ja
				Für 18 h (Empfehlung 2 Spritzen für je 12 h)
Metamizol	50 mg/mL	2500 mg/50 mL	0,9 % NaCl	Ja
				Bei Gelbfärbung: kein wesentlicher Aktivitätsverlust
Midazolam ^4^	2 mg/mL	100 mg/50 mL	Entfällt	Ja
Milrinon	0,2 mg/mL	10 mg/50 mL	0,9 % NaCl, G5 %	Ja
Morphinsulfat	1 mg/mL	50 mg/50 mL	0,9 % NaCl, G5 %	Ja
Natriumchlorid	1 mmol/mL Na^+^/1 mmol/mL Cl^−^	50 mmol/50 mmol/50 mL	Entfällt	Ja
Natriumphosphat ^5^	1 mmol/mL Na^+^/	50 mmol/30 mmol/50 mL	Entfällt	Ja
	0,6 mmol/mL PO4^3−^			
Nimodipin	0,2 mg/ml	10 mg/50 mL	Entfällt	Ja
				Wenn Lichtschutz bei Infusionsdauer > 10 h vorhanden
Norepinephrin	0,02 mg/mL	1 mg/50 mL	G5 %, 0,9 % NaCl	Ja
Norepinephrin	0,1 mg/mL	5 mg/50 mL	G5 %, 0,9 % NaCl	Ja
Norepinephrin	0,2 mg/mL	10 mg/50 mL	G5 %, 0,9 % NaCl	Ja
Norepinephrin	0,4 mg/mL	20 mg/50 mL	G5 %, 0,9 % NaCl	Ja
Piperacillin/Tazobactam	80 mg/mL Piperacillin/10 mg/mL Tazobactam	4000 mg/500 mg/50 mL	0,9 % NaCl, G5 %	Ja
Propofol	20 mg/mL	1000 mg/50 mL	Entfällt	Ja
				Max. 12 h wg. mikrobiolog. Instabilität
Remifentanil	0,1 mg/mL	5 mg/50 mL	0,9 % NaCl	Ja
Sufentanil	5 µg/mL	250 µg/50 mL	Entfällt	Ja
Sufentanil	10 µg/mL	500 µg/50 mL	Entfällt	Ja
Sufentanil	20 µg/mL	1000 µg/50 mL	0,9 % NaCl, G5 %	Ja
Terlipressinacetat	0,04 mg/mL	2 mg/50 mL	0,9 % NaCl	Ja
Tirofiban	0,05 mg/mL	12,5 mg/250 mL Infusionsbeutel	Entfällt	Ja
Urapidil	5 mg/mL	250 mg/50 mL	Entfällt	Ja
Vancomycin	20 mg/mL	1000 mg/50 mL	0,9 % NaCl	Ja
Vasopressin/Argipressin	0,8 I.E./mL	40 I.E./50 mL	0,9 % NaCl	Ja

^1^ Amiodaron als Ready-To-Use-Anwendung (20 mg/mL) steht derzeit nicht auf dem deutschen Markt zu Verfügung

^2^ bei Lieferschwierigkeiten kann Ketamin (50 mg/mL) zu gleichen Laufraten eingesetzt werden

^3^ Magnesiumsulfat-Heptahydrat 1000 mg entsprechen: Mg^2+^ 4,05 mmol = Mg^2+^ 98,63 mg

^4^ Midazolam 1 mg/mL, 50 mL als Fertigarzneimittel verfügbar, kann alternativ zum Einsatz kommen

^5^ Alternativ kann Natrium-Glycerophosphat (2 mmol/ml Na^+^ und 1 mmol/mL PO_4_^3−^) zum Einsatz kommen

* Zugrunde liegende Originalliteratur siehe https://www.divi.de/publikationen/alle-publikationen/sektionen/angewandte-pharmakologie-in-der-notfall-und-intensivmedizin bzw. https://www.adka.de/news/details/empfehlungen-zu-standardkonzentrationen-fuer-die-kontinuierliche-infusion-auf-intensivstationen

Wenn ein zugelassenes Fertigarzneimittel oder in der Krankenhausapotheke eigenhergestelltes Arzneimittel verfügbar ist, wurde unter dem Aspekt der AMTS und Effizienz bevorzugt die verfügbare Konzentration ausgewählt. Zur Vermeidung des unbeabsichtigten Überschreitens der Tagesmaximaldosis bei Metamizol von 4000 mg (bei Bedarf 5000 mg) wurde die Konzentration 50 mg/mL empfohlen [1]. Aus Sicherheitsgründen wurde auch bei Clonidin und Dihydralazin die niedrigere der vorgeschlagenen Konzentrationen als SK gewählt. Aus der vierwöchigen Kommentierungsphase durch DIVI-Mitglieder resultierten zwei Ergänzungen (s. Amiodaron, Midazolam), die als Fußnoten in die SKL aufgenommen wurden.

In der verabschiedeten nationalen ADKA/DIVI-SKL ist der Wirkstoff mit der ausgewählten Standardkonzentration, Standardmenge/-volumen, der Trägerlösung und Angaben zur physikalisch-chemischen Stabilität (max. 24 h, RT) gelistet (Tab. 3). Die Angaben zur physikalisch-chemischen Stabilität gelten für die ausgewählten Trägerlösungen, die festgelegte Konzentration und RT. Sie können nicht ungeprüft auf andere Bedingungen (z. B. andere Trägerlösung, Konzentration, Temperatur, Sauerstoff‑/Lichtverhältnisse, Primärpackmittel) übertragen werden [4]. Die meisten Wirkstoffe sind in der angegebenen Konzentration physikalisch-chemisch stabil (max. 10 % Gehaltsabnahme im Vergleich zum Anfangsgehalt). Ausnahmen sind Meropenem und Alteplase. Für Meropenem 20 mg/mL in NaCl 0,9 % ist bei RT physikalisch-chemische Stabilität für 18 h nachgewiesen, daraus resultiert ein praxistaugliches Wechselintervall von 12 h. Für Alteplase ist physikalisch-chemische Stabilität für 8 h nachgewiesen (Tab. 3).

Die Etablierung der SK muss als interprofessionelles Projekt klinikweit erfolgen. Werden noch keine Standards genutzt, wird eine zeitnahe Implementierung mit Schulungsangeboten empfohlen. Sind bereits klinikeigene SKL etabliert, sollten Anpassungen an die bundesweiten SK sorgfältig geplant und ggf. schrittweise umgesetzt werden [18].

## Diskussion

Die bundesweit gültige SKL zur geschwindigkeitsgesteuerten kontinuierlichen Infusion mit Standardkonzentrationen auf Intensivstationen wurde von einer mandatierten interdisziplinären Expertengruppe in einem zweistufigen Verfahren literaturgestützt und konsensbasiert entwickelt. Als Grundlage dienten die Ergebnisse einer DIVI-Umfrage sowie nationale SKL aus UK und USA [5, 19, 27]. Die Entscheidungskriterien zum Einschluss oder Ausschluss eines Arzneimittels bzw. einer SK beinhalten primär Sicherheitsaspekte. In Fällen, in denen die individuelle Dosis aus den zeitgleich gemessenen Vital- und Laborparametern resultiert, kann die Dosisanpassung über die Laufgeschwindigkeit der Infusion einfach und sicher erreicht werden [8].

### Wirkstoffauswahl

Die Auswahl der in die SKL aufzunehmenden Wirkstoffen folgte dem Prinzip ‚so schmal wie möglich, so breit wie nötig‘ und stellt einen pragmatischen Ansatz dar. Die gewählten Ausschlusskriterien (*seltene Anwendung, fraglicher Nutzen einer Dauerinfusion im Vergleich zur Kurzinfusion, Off-Label-Anwendung)* sind häufig überlappend zutreffend. Wenn die Dauerinfusion eine Off-Label-Anwendung darstellt, z. B. bei Metoprolol und Dalteparin, wird diese seltener eingesetzt.

Um Verwechslungen zu vermeiden, wurde auf peridural applizierte Arzneimittel/-Kombinationen verzichtet. Analog zu den USA [5] wird dafür zukünftig eine bundesweite, spezifische SKL angestrebt. Als für in Deutschland übliche Wirkstoffe sind Alprostadil, Dihydralazin, Esketamin, Hydrocortison, Metamizol, Nimodipin und Urapidil enthalten. Mit insgesamt 41 eingeschlossenen Arzneimitteln ist die erarbeitete SKL umfangreicher als die USA-Liste mit 36 Arzneimitteln und die UK-Liste mit 19 Arzneimitteln [5, 27]. Für Wirkstoffe, die in allen drei nationalen Listen vorhanden sind, gibt es große Übereinstimmungen in den gewählten SK (vgl. Tabelle S1 im Online-Zusatzmaterial).

Im Unterschied zur USA-Liste sind Antibiotika, die prolongiert oder kontinuierlich appliziert werden und konzentrierte Elektrolytlösungen zur volumensparenden Applikation gelistet (Kaliumchlorid, Natriumchlorid, Natriumphosphat, Magnesiumsulfat). Die prolongierte/kontinuierliche Applikation von Beta-Lactam-Antibiotika verbessert die Heilungsraten, ohne dass die Toxizität zu nimmt [7, 12]. Für Vancomycin wird postuliert, dass die Dauerinfusion nierenverträglicher und die Einstellung der therapeutischen Wirkspiegel vereinfacht ist [7].

Für **hochkonzentrierte Elektrolytkonzentrate** ist die Applikation über Pumpensysteme sowie die Standardisierung der Bezeichnung, der Dosis und Maßeinheit (z. B. mol) empfohlen [2, 3, 21]. Die kontinuierliche Applikation von unverdünnter 1‑molarer Kaliumchlorid-Lösung entspricht nicht dem Zulassungstext des Fertigarzneimittels, ist aber indiziert bei Patienten mit sehr hohem Kaliumbedarf. Bei Beachtung der 2023 veröffentlichten Handlungsempfehlung des Aktionsbündnisses Patientensicherheit ist Applikationssicherheit gewährleistet [3]. Analog wurden für weitere Elektrolytkonzentrate, so für Magnesiumsulfat mit 0,4 mmol/mL Mg^2+^-Ionen, einheitliche und praxistaugliche SK festgelegt [15].

### Anwendung der Standardkonzentration

Die Nutzung von Smart-Infusionspumpen mit integrierten Arzneimittelbibliotheken gehört zu den wirksamen Maßnahmen, um Applikationsfehler zu vermeiden [11]. Die Erstellung von Arzneimittelbibliotheken erfordert Standardkonzentrationen und -volumen [11, 28]. Die Expertengruppe wählte als SK jeweils eine für die überwiegende Zahl von Patienten angemessene Konzentration [5]. So wurden bewusst teils niedrigere Konzentrationen gewählt, um geringe Laufraten und/oder zu lange Standzeiten (z. B. Morphinsulfat, Clonidin) zu vermeiden. Sehr niedrige Laufraten können durch längere Anlaufzeiten und Unterbrechungen beim Spritzenwechsel zu einer verzögerten und unregelmäßigen Arzneimittelwirkung führen. Darüber hinaus kann die Zeit bei kleinen Laufraten bis zu einem Okklusionsalarm verlängert sein [23, 28]. Andererseits beinhalten auch zu häufige Spritzenwechsel ein Fehlerrisiko. Für einzelne Patientengruppen kann es sinnvoll sein, von den Empfehlungen abzuweichen und andere Konzentrationen einzusetzen. Ein Beispiel ist die Argatroban-SK (1 mg/mL), die dem applikationsfertigen Fertigarzneimittel entspricht, aber bei leberinsuffizienten Patienten zu (sehr) niedrigen Laufraten führen würde. Zur Festlegung der SK wurden auch die Wirkstoffmengen und Konzentrationen in den verfügbaren Fertigarzneimitteln und die Anzahl der Zubereitungsschritte berücksichtigt. Für Amiodaron (21 mg/mL) wurde ein sinnvolles Vielfaches der verfügbaren Fertigarzneimittelampullen gewählt. In wenigen Ausnahmen wird die Applikation größerer Volumen als 50 mL wegen der Verfügbarkeit applikationsfertiger Infusionslösungen (Tirofiban, Esmolol) oder wegen Instabilität der konzentrierten Lösung (z. B. Opaleszenz und Ausfällung bei Levosimendan-Konzentration > 0,05 mg/mL) bevorzugt (Tab. 3). Anderseits wird in der Praxis bei vorhandener Flüssigkeitsrestriktion auf konzentrierte Lösungen ausgewichen (z. B. Levosimendan 0,25 mg/mL), auch wenn die physikalisch-chemische Stabilität nicht abschließend nachgewiesen ist.

### Stabilität der applikationsfertigen Arzneimittelinfusionen

Die sichere Applikation einer Arzneimittelinfusion setzt deren mikrobiologische und physikalisch-chemische Stabilität voraus. Wenn die Vorbereitung der Infusionslösung im Stationsbereich erfolgt, sollen die applikationsfertigen Lösungen nicht länger als eine Stunde im Voraus rekonstituiert werden, um das Risiko der Keimvermehrung im Falle einer akzidentellen mikrobiologischen Kontamination zu begrenzen [22]. Aus dem gleichen Grund sollen die Infusionslösungen innerhalb von 24 h appliziert bzw. Reste danach verworfen werden [10]. Wegen der wachstumsfördernden Eigenschaften dürfen reine Lipidemulsionen und Propofol-Infusionen maximal 12 h nach Anbruch appliziert werden. Danach müssen Infusionslösung und Infusionsleitung getauscht werden (s. auch Tab. 3; [22]). Standardkonzentrationen und die standardisierten sonstigen stabilitätsdeterminierenden Faktoren (u. a. Trägerlösung, Temperatur) erlauben eine sichere Bewertung der physikalisch-chemischen Stabilität der applikationsfertigen Infusionslösung über 24 h mit Hilfe der Fachinformation und/oder Originalpublikationen. Wenn mehrere Referenzen vorlagen, wurde diejenige mit der höchsten Evidenz als Informationsgrundlage gewählt.

### Limitationen

Zur Erstellung der SKL wurden wenige Experten durch die Fachgesellschaften DIVI und ADKA mandatiert. Da die Empfehlungen sehr stark evidenzbasiert auf einer aktuellen Umfrage unter deutschen Intensivmedizinern und USA- und UK-Empfehlungen basieren, hätte eine größere Expertenzahl wahrscheinlich nur zu minimal anderen Ergebnissen geführt. Außerdem war der mehrstufige Entscheidungsprozess transparent und stringent, sodass weitere Experten die Konsensfindung wahrscheinlich nicht wesentlich verändert hätten. Ein intensiverer Hinweis auf die Kommentierungsmöglichkeit, insbesondere durch Pflegefachpersonen, hätte möglicherweise weitere Verbesserungen ergeben. Wesentliche Rückmeldungen und Anmerkungen in der Folge dieser Publikation sollten Anlass für eine zeitnahe Revision sein. Es gab keine Beteiligung aus der pharmazeutischen Industrie, damit wurde eine wirtschaftlich motivierte Entscheidungsfindung minimiert bzw. ausgeschlossen. Andererseits wäre es hilfreich, wenn die pharmazeutische Industrie eingebunden und vermehrt Fertigarzneimittel in den gewählten SK auf den Markt bringen würde.

Eine größere Zahl von kontinuierlich infundierten Arzneimitteln wurde nicht in die Liste aufgenommen, und einige Anwender mögen Empfehlungen dazu vermissen. Doch wurde großes Augenmerk darauf gerichtet, in Deutschland häufig kontinuierlich applizierte Arzneimittel zu identifizieren, und die verabschiedete SKL ist umfangreicher als andere nationale SKL.

Die vorliegenden Empfehlungen sind nicht verbindlich. Sie mögen nicht für jede Klinik, jede Intensivstation oder jeden Patienten am besten geeignet sein. Individuelle (krankenhausinterne) Bedingungen wie ein bestimmtes Patientenkollektiv, Lieferengpässe oder individuelle Sicherheitsbedenken können abweichende Dosierungen begründen. Das Risiko für Medikationsfehler durch fehlende Standardisierung ist aber höher, insbesondere, wenn neue Mitarbeiter aus dem ärztlichen oder pflegerischen Bereich ihre Tätigkeit aufnehmen. Zudem sind auch Personalwechsel zwischen Intensivstationen (z. B. zur Aushilfe bei Engpässen) oder der Einsatz von Leihkräften als Risikofaktoren anzusehen.

Bei einer Umstellung auf eine der empfohlenen Dosierungen muss dem üblichen Widerstreben gegen Veränderung durch ein geeignetes ‚change management‘ entgegengewirkt werden. Eine Änderung der gewohnten Abläufe, so auch die Integration der SKL in den Medikationsprozess auf den Intensivstationen, bringt naturgemäß Fehlerrisiken mit sich. Die Neuerungen müssen geschult und trainiert, neu auftretende Fehler und/oder Risiken analysiert werden. In regelmäßigen Abständen muss eine Anpassung an die medizinischen Entwicklungen, aber auch an den Arzneimittelmarkt erfolgen. In einem angemessenen Zeitabstand sind eine Akzeptanzanalyse und Aktualisierungen der SKL vorzusehen.

## Schlussfolgerung

Die von der ADKA/DIVI-Expertengruppe entwickelten Empfehlungen zu Standardkonzentrationen für die kontinuierliche Infusion sind praxisnah und spezifisch für die in Deutschland zur Behandlung von Intensivpatienten relevanten Wirkstoffe. Die evidenz- und konsensbasierte SKL soll bundesweit auf Erwachsenen-Intensivstationen etabliert werden, um eine sichere, wirksame und effiziente Arzneimitteltherapie zu gewährleisten und Medikationsfehler zu vermeiden.

## Fazit für die Praxis

- Erstmals wurde eine bundesweit gültige Standardkonzentrationsliste (SKL) für die Intensivstationen von einer interprofessionellen Expertengruppe entwickelt.
- Die konsentierte Liste enthält 41 Wirkstoffe mit 49 Standardkonzentrationen (SK).
- Die SKL für Dauerinfusionen stellt einen wesentlichen Baustein zur Verbesserung der Arzneimitteltherapiesicherheit (AMTS) dar.
- Die Implementierung der SKL soll von einem interprofessionellen Team begleitet werden.

## Supplementary Information

Tabelle S1: Übersicht über aufgenommene und ausgeschlossene Standardkonzentrationen nach Expertendiskussion in der Stufe 2.

## Abkürzungen

0,9 % NaCl: 0,9 %ige Natriumchlorid-Lösung
G5 %: 5 %ige Glukoselösung
I.E.: Internationale Einheit
PP: Polypropylen
RT: Raumtemperatur
