# Supplementary material for: Recommendations on standard concentrations for continuous infusion of medicinal products in intensive care units
Source: Med Klin Intensivmed Notfmed. 2025 Mar 20;120(3):199–207. [Article in German] doi: 10.1007/s00063-025-01264-x (PMC11961480; doi:10.1007/s00063-025-01264-x)
Supplement: Supplementary file 1 — Tabelle S1: Übersicht über aufgenommene und ausgeschlossene Standardkonzentrationen nach Expertendiskussion in der Stufe 2. [file 63_2025_1264_MOESM1_ESM.pdf]

Tabelle S1: Übersicht über aufgenommene und ausgeschlossene Standardkonzentrationen nach Expertendiskussion in der Stufe 2

Wirkstoff	Standardkonzentration	Akzeptanzrate für Konzentration gemäß DIVI-Umfrage [%] [19]	Empfehlung in nationalen Standardkonzentrationslisten		Empfehlung in ausgewählter Literatur		Verfügbarkeit applikations-fertiger FAM	Einschluss	Entscheidungs-kriterium/ Begründung
			UK 2020 [27]	USA 2024 [5]	UCL Hospitals Injectable Medicines [17]	Pocket Guide Intensivmedizin [6]			
Alprostadil	0,8 µg/mL	26	-	-	-	-	-	Ja	-
Alprostadil	1,2 µg/mL	46	-	-	-	-	-	Nein	S2-3
Alteplase	1 mg/mL	93	-	1 mg/mL	1 mg/mL	1; 2 mg/mL	Ja	Ja	-
Amiodaron	6 mg/mL	25	0,6; 1,2; 1,8; 6; 12; 18 mg/mL	1,8 mg/mL	-	18 mg/mL	Nein*	Nein	S2-3
Amiodaron	18 mg/mL	28						Nein	S2-3
Amiodaron	20 mg/mL	-						Nein	S2-3
Amiodaron	21 mg/mL	8						Ja	-
Amiodaron	24 mg/mL	36						Nein	S2-3
Argatroban	0,5 mg/mL	32	-	1 mg/mL	-	-	-	Nein	S2-3
Argatroban	1 mg/mL	47					Ja	Ja	-
Ceftazidim	40 mg/mL	36	-	-	-	-	-	Ja	-
Ceftazidim	80 mg/ml	58					-	Nein	S2-3
Clonidin	15 µg/mL	20	15 µg/mL	-	20 µg/mL	15 µg/mL	-	Ja	-
Clonidin	30 µg/mL	67					-	Nein	S2-3, Risiko Überdosierung
Dexmedetomidin	8 µg/mL	67	4; 8 µg/mL	4 µg/mL	-	4; 8 µg/mL	-	Ja	-
Dexmedetomidin	20 µg/mL	26					-	Nein	S2-3
Dihydralazin	1 mg/mL	38	-	-	-	-	-	Ja	-
Dihydralazin	2 mg/mL	53					-	Nein	S2-3, Risiko Überschreitung Tagesmaximaldosis
Dobutamin	5 mg/mL	91	5 mg/mL	4 mg/mL	5 mg/mL	5 mg/mL	Ja	Ja	-
Epinephrin	0,02 mg/mL	20	-	0,02 mg/mL (250 mL)	0,04 mg/mL	-	-	Ja	-
Epinephrin	0,1 mg/mL	70	0,08 mg/mL	0,04 mg/mL (250 mL)	0,08 mg/mL	0,1 mg/mL	-	Ja	-
Epinephrin	0,2 mg/mL	47	0,16 mg/mL	-	0,16 mg/mL	-	-	Ja	-
Epoprostenol	2 µg/mL	100	-	-	10 µg/mL	-	-	Ja	-
Esketamin	25 mg/mL	84	-	-	-	25 mg/mL	Ja	Ja	-
Esmolol	10 mg/mL (250mL)	93	10; 20 mg/mL	10 mg/mL	10 mg/mL	10; 50 mg/mL	Ja	Ja	-

Flucloxacillin	80 mg/mL	-	-	-	-	-	-	Ja	-
Furosemid	10 mg/mL	68	-	2; 10 mg/mL	-	5 mg/mL	-	Ja	-
Glyceroltrinitrat	1 mg/mL	96	-	0,2 mg/mL	1 mg/mL	0,1; 0,2; 0,5; 1 mg/mL	Ja	Ja	-
Heparin	200 I.E./mL	46	-	-	-	100; 200 I.E./mL	-	Ja	-
Heparin	500 I.E./mL	43	1000 I.E./mL	100 I.E./mL (250 mL)	1000 I.E./mL	500 I.E./mL	-	Ja	-
Hydrocortison	2 mg/mL	68	-	-	-	1 mg/mL	-	Ja	-
Iloprost	2 µg/mL	78	-	-	1 µg/mL	-	-	Ja	-
Insulin (human)	1 I.E./mL	94	1 I.E./mL	1 I.E./mL	1 I.E./mL	1 I.E./mL	-	Ja	-
Kaliumchlorid	1 mmol/mL	98	-	-	0,4 mmol/mL	1 mmol/mL	Ja	Ja	-
Ketamin	20 mg/mL	62	-	2; 10 mg/mL	-	-	-	Nein	S2-3
Ketamin	50 mg/mL	23	-	-	50 mg/mL	-	-	Ja	-
Levosimendan	0,025 mg/mL (500 mL)	52	0,05 mg/mL	-	-	0,025; 0,05 mg/mL	-	Ja	-
Levosimendan	0,25 mg/mL	50					-	Nein	S2-6
Magnesiumsulfat	0,4 mmol/mL (Mg^{2+})	-	0,4 mmol/mL	0,16 mmol/mL	0,4 mmol/mL	(0,02-0,05); 1,6 mmol/mL	-	Ja	-
Meropenem	20 mg/mL	-	10; 20 mg/mL	-	-	-	-	Ja	-
Metamizol	50 mg/mL	7	-	-	-	-	-	Ja	-
Metamizol	100 mg/mL	79	-	-	-	-	-	Nein	S2-3, Risko Überschreitung Tagesmaximaldosis
Midazolam	2 mg/mL	19	1; 2 mg/mL	1 mg/mL	2 mg/mL	1; 2 mg/mL	Ja	Ja	-
Milrinon	0,2 mg/mL	90	0,2 mg/mL	0,2 mg/mL	0,2 mg/mL	-	-	Ja	-
Morphin-sulfat	1 mg/mL	41	1; 2 mg/mL	1; 5 mg/mL	1 mg/mL	1; 0,2 mg/mL	-	Ja	-
Morphin-sulfat	2 mg/mL	56					-	Nein	S2-3
Natriumchlorid	1 mmol/mL (5,85%)	89	-	-	-	-	Ja	Ja	-
Natriumphosphat	1 / 0,6 mmol/mL (Na^+/ PO_4^{3-})	86	-	-	0,1/ 0,06 mmol/mL	-	-	Ja	-
Nimodipin	0,2 mg/ml	96	-	-	-	-	Ja	Ja	-
Norepinephrin	0,02 mg/mL	9	-	-	-	-	-	Ja	-
Norepinephrin	0,1 mg/mL	72	0,8 mg/mL	0,016 (250 mL)	0,8 mg/mL	0,1 mg/mL	Ja	Ja	-
Norepinephrin	0,2 mg/mL	64	0,16 mg/mL	0,032 mg/mL (250 mL)	0,16 mg/mL	0,2 mg/mL	Ja	Ja	-
Norepinephrin	0,4 mg/mL	17	0,32 mg/mL	0,128 mg/mL (250 mL)	-	-	-	Ja	-
Piperacillin/ Tazobactam	80 / 10 mg/mL	97	80 / 10 mg/mL	-	80 / 10 mg/mL	-	-	Ja	-
Propofol	20 mg/mL	91	10 mg/mL	-	20 mg/mL	20 mg/mL	Ja	Ja	-

Remifentanil	0,1 mg/mL	79	0,05; 0,1 mg/mL	-	0,1 mg/mL	0,05; 0,1 mg/mL	-	Ja	-
Sufentanil	5 µg/mL	20	-	-	-	-	-	Ja	-
Sufentanil	10 µg/mL	58	-	-	-	-	Ja	Ja	-
Sufentanil	20 µg/mL	10	-	-	-	20 µg/mL	-	Ja	-
Terlipressinacetat	0,04 mg/mL	-	-	-	0,12 mg/mL	0,1; 0,2 mg/mL	-	Ja	-
Tirofiban	0,05 mg/mL (250 mL)	94	-	-	-	0,02; 0,05 mg/mL	Ja	Ja	-
Urapidil	5 mg/mL	84	-	-	-	-	-	Ja	-
Vancomycin	20 mg/mL	-	-	-	5 mg/mL	-	-	Ja	-
Vancomycin	40 mg/mL	-	-	-	5 mg/mL	-	-	Nein	S2-3,<br>zu niedrige Laufrate bei Dosisanpassung
Vasopressin/ Argipressin	0,8 I.E. /mL	-	0,4 I.E./mL	0,2; 0,4;<br>1 I.E./mL	0,4 I.E./mL	-	-	Ja	-

*Kurzzeitige Verfügbarkeit 2023, z.Z. nicht verfügbar.
